# Opportunities for linking research to policy: lessons learned from implementation research in sexual and reproductive health within the ANSER network

**DOI:** 10.1186/s12961-018-0397-7

**Published:** 2018-12-17

**Authors:** ANSER, Emilomo Ogbe, Dirk Van Braeckel, Marleen Temmerman, Elin C. Larsson, Ines Keygnaert, Wilson De los Reyes Aragón, Feng Cheng, Gunta Lazdane, Diane Cooper, Simukai Shamu, Peter Gichangi, Sónia Dias, Hazel Barrett, Anne Nobels, Kaiyan Pei, Anna Galle, Tammary Esho, Lucia Knight, Hanani Tabana, Olivier Degomme

**Affiliations:** 1 International Centre for Reproductive Health, Ghent University, Ghent, Belgium; 2 Ghent University, Aga Khan University, Nairobi, Kenya; 3 Department of Women’s and Children’s Health, Uppsala University/Karolinska Institutet, Uppsala, Sweden; 4 RFSU - the Swedish Association for Sexuality Education, Stockholm, Sweden; 5 School of Medicine and Research Centre for Public Health, Tsinghua University, Beijing, China; 6 Department of Obstetrics and Gynaecology, Riga Stradins University, Riga, Latvia; 7 School of Public Health, University of the Western Cape, Cape Town, South Africa; 8 Foundation for Professional Development, Pretoria, South Africa; 9 University of Nairobi, Ghent University and International Centre for Reproductive Health, Nairobi, Kenya; 10 Escola Nacional de Saúde Pública, Centro de Investigação em Saúde Pública, Universidade NOVA de Lisboa, Lisbon, Portugal; 11 Centre for Trust, Peace and Social relations, Coventry University, Coventry, United Kingdom; 12 National Research Institute for Family Planning, Beijing, China; 13 Department of Community and Public Health, Technical University of Kenya, Nairobi, Kenya; 14 School of Public Health, University of the Witwatersrand, Johannesburg, South Africa

**Keywords:** Sexual and reproductive health, research, health policy, global health, ANSER

## Abstract

**Background:**

The uptake of findings from sexual and reproductive health and rights research into policy-making remains a complex and non-linear process. Different models of research utilisation and guidelines to maximise this in policy-making exist, however, challenges still remain for researchers to improve uptake of their research findings and for policy-makers to use research evidence in their work.

**Methods:**

A participatory workshop with researchers was organised in November 2017 by the Academic Network for Sexual and Reproductive Health and Rights Policy (ANSER) to address this gap. ANSER is a consortium of experienced researchers, some of whom have policy-making experience, working on sexual and reproductive health and rights issues across 16 countries and 5 continents. The experiential learning cycle was used to guide the workshop discussions based on case studies and to encourage participants to focus on key lessons learned. Workshop findings were thematically analysed using specific stages from Hanney et al.’s (*Health Res Policy Syst* 1:2, 2003) framework on the place of policy-making in the stages of assessment of research utilisation and outcomes.

**Results:**

The workshop identified key strategies for translating research into policy, including joint agenda-setting between researchers and policy-makers, as well as building trust and partnerships with different stakeholders. These were linked to stages within Hanney et al.’s framework as opportunities for engaging with policy-makers to ensure uptake of research findings.

**Conclusion:**

The engagement of stakeholders during the research development and implementation phases, especially at strategic moments, has a positive impact on uptake of research findings. The strategies and stages described in this paper can be applied to improve utilisation of research findings into policy development and implementation globally.

## Introduction

The impact of research on policy development is complex [1]. Policy formulation and implementation processes do not necessarily incorporate knowledge and research evidence, while research findings do not necessarily result in policy changes [1, 2]. Research plays a vital role in providing evidence for individual and social interventions that have the potential to impact on healthcare delivery and utilisation in different health systems. It could also provide feasible cost-effective solutions by synthesising evidence of ‘what works best’, ‘for whom’ and ‘in which contexts’ [3]. Weiss discussed the different models of research utilisation and grouped them into ‘the knowledge-driven model’, ‘the problem solving model’, ‘the interactive model’, ‘the political model’, ‘the tactical model’ and ‘the enlightenment model’ and defined ‘research as part of the intellectual enterprise of the society’ [4]. These different models explain the spectrum of research utilisation by policy-makers, going from a linear process (knowledge-driven model) that assumes uptake of evidence is based on the existence of information and relevant technology only, to more dynamic interactive models that take into account context, political priorities, stakeholder involvement and multiple sources of information used in the policy development process [4].

The uptake of research findings in policy development remains challenging, as this process is influenced by a myriad of societal factors, including the availability of resources, values of the policy-makers and the socio-political context [5]. These difficulties are not taken into account in the development and implementation of research. Researchers often believe that, if their research is rigorous enough and the findings are published, uptake by politicians and service providers would be inevitable [6]. However, in reality, this is rarely the case. The existence of published, relevant evidence-based research is not sufficient to ensure uptake [7]. Translating research evidence into policy involves an emotive component, of manipulation and persuasion, that most researchers are either ignorant of or unwilling to do; however, this is essential to framing the policy dialogue [8].

There are several challenges to translating research into policy in the health policy environment, including the ‘dynamic nature of the health policy environment’, and the fact that health policy is interwoven with other domains. These challenges make documenting evidence and navigating the different interests of policy-makers and other stakeholders difficult [9]. In this article, by ‘stakeholders’ we refer to the different actors involved in sexual and reproductive health and rights (SRHR) policy formulation, namely policy-makers and civil society. A systematic review of barriers and facilitators to uptake of research evidence by policy-makers identified *“unavailability or lack of access to research evidence, level of clarity/relevance/reliability of research eveidence, lack of time or opportunity to utilise research evidence, costs and lack of knowledge of research methods*” as commonly cited barriers to research uptake [10]. Facilitators commonly cited as important were *“timely access to good quality and relevant research evidence, and collaborations between researchers and policy-makers, as the most important factors that influence the uptake of evidence by policy-makers*” [10]. These findings address some of the inherent assumptions made in existing health policy research about policy-makers and what ‘evidence-based policy-making’ means. Policy-makers will prioritise the perceived relevance of the research evidence to their policy strategy over the values attached to research methodology and quality by researchers. A critique of assumptions that portray policy-makers as “*interest-oriented and indifferent to evidence*” calls to attention the need for nuance in interpreting research on uptake of evidence by policy-makers [8]. Oliver et al. [8, 10] encourage a shift in focus from “*evidence of research uptake*” to understanding the value placed on specific sources and types of information by policy-makers, for example, locally sourced data might be more valued than randomised controlled trials that are published and recognised internationally.

In the field of sexual and reproductive health (SRH) research, as some interventions are targeted at vulnerable groups and at topics that are often culturally sensitive, difficulties emerge in navigating conflicts between research evidence and cultural and political norms or debates. Policy-makers have competing priorities that are value laden and influenced by context, requiring the added effort of advocacy for “*SRH supportive policies*” as a way to encourage prioritisation of SRH policies [11].

The Academic Network for Sexual and Reproductive Health and Rights Policy (ANSER) was developed to address the gap between research and policy in SRHR. It is a global platform for SRHR policy research, education and healthcare delivery. The network does so by initiating collaborative research on SRHR policy-related topics, by developing a portfolio of education and training programmes on SRHR policy, and by fostering interaction between SRHR researchers and policy-makers. The network is currently composed of 28 institutions in 16 countries, across 5 continents. A workshop was organised to pool the knowledge of ANSER member experts on best practices for translating SRH research into policies. The workshop took place on the November 29, 2017, at Ghent University, Belgium. Selected case studies were presented and interactive group discussions held to develop recommendations for promoting translation of SRH research into policy. This article presents some of the main conclusions from the workshop and strategies to ensure research uptake.

## Methods

A facilitated interactive workshop within the ANSER was organised, incorporating an experiential learning cycle developed by Kolb et al. [12] to ensure that discussions were reflective and based on key learning points from the experiences of the researchers present. Three case studies, which were based on successful methods that promoted the ‘uptake of research evidence’ into policy development and implementation, were presented by experts. The 22 workshop participants included SRH researchers from different leading academic institutions based in South Africa, Germany, China, Kenya, Nigeria, Portugal, Belgium and the United Kingdom, as well as SRHR experts with experience in developing international health policies drawn from different continents, including the European Union, Africa and Asia.

The presenters for the workshop included Gunta Lazdane (former Programme Manager, Sexual and Reproductive Health, WHO Regional Office for Europe) who introduced key issues around translating SRHR research into policy based on her experience working at the WHO Regional Office in Europe. Case studies were then presented by Marleen Temmerman (Gynaecologist, Professor, former Director of the Department of Reproductive Health and Research, at WHO and former Senator), Ines Keygnaert (Senior Researcher at Ghent University responsible for implementing the first National Programme for Sexual Assault Care Centres in Belgium) and Wilson de los Reyes (Senior Legal Advisor and Representative to the UN for The Swedish Association for Sexuality Education – The Swedish Association for Sexuality Education). The case studies covered experiences of implementing policies influenced by SRHR research in the European Union, Africa and Asia.

These experts’ presentations outlined the best practices and key learning points from their experiences; these were discussed in three groups, using facilitated interactive methods. Participants were randomly assigned to the groups to ensure a diverse representation of interests and experiences. The reflexive discussions were guided by questions focused on identifying the significant changes that resulted from the programme, and implications of their research for policy implementation. More details about these research questions are included in the Appendix. The workshop participants proposed concrete ‘real world’ applications of the lessons learned.

**Fig. 1:**
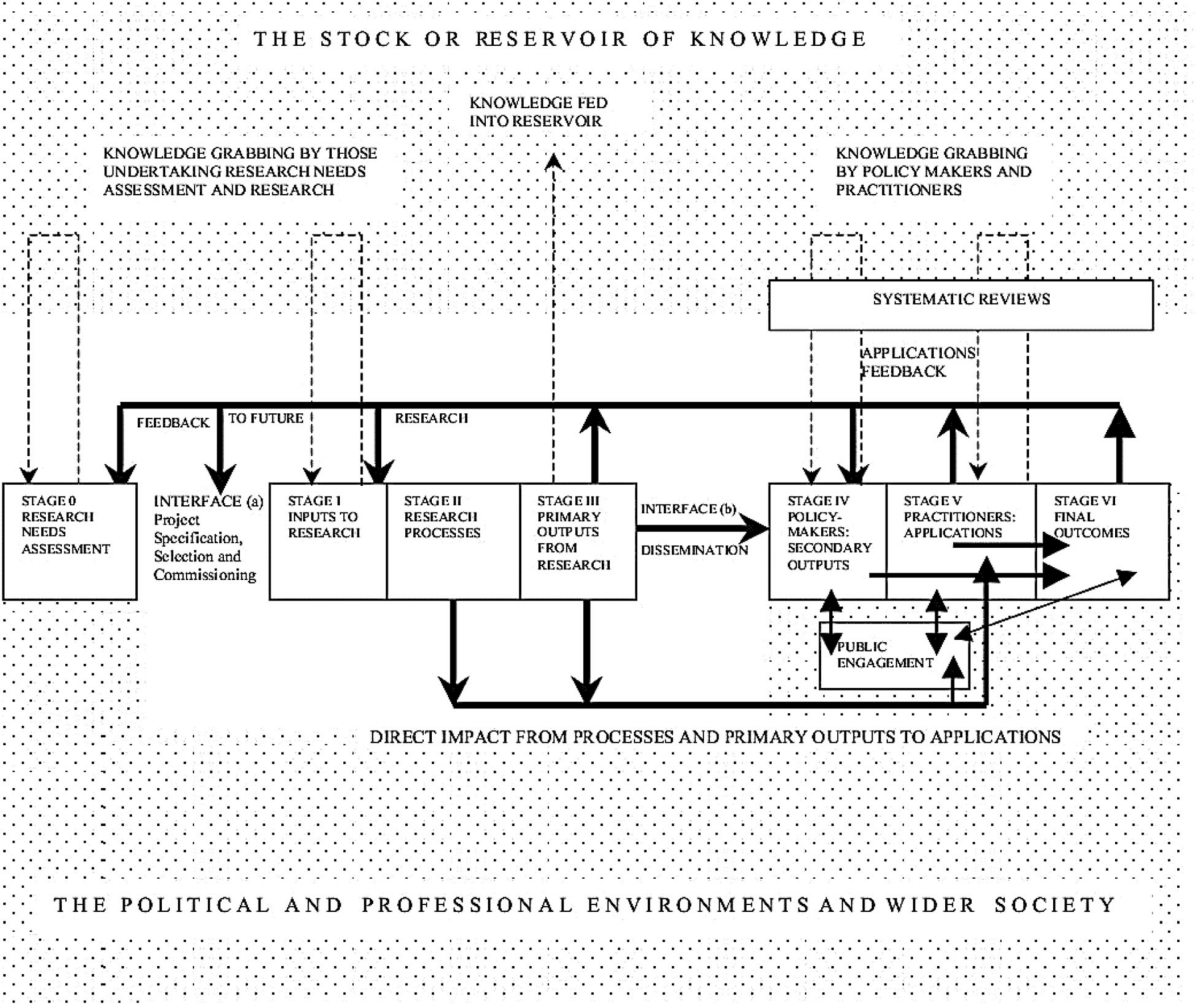
The place of policy-making in the stages of assessment of research utilisation and outcomes [1]

The results of the interactive sessions were documented and key recommendations outlined. Key themes and discourses were identified and linked with recommendations focused on how researchers can engage with stakeholders and work together more efficiently. The findings were interpreted using the framework proposed by Hanney et al. [1], which outlines the “*place of policy-making in the stages of assessment of research utilisation and final outcomes*” (Fig. 1, [1]). Key stages relevant to the case studies were identified within the framework and used for this purpose.

The key stages relevant to the case studies are outlined below:

- Stage 0: At the point of research needs assessment
- Stage 1: Providing input to research assessment
- Stage 4: Dissemination of research findings
- Stage 5: Application of secondary outputs of research in engagement with practitioners and other stakeholders

### Case study summaries

#### Case study 1: Instituting comprehensive sexuality education (CSE) in schools (Stages 1, 4, 5)

This project involved a collaboration between the University of Maastricht and the Youth Harvest Foundation, followed by reporting of research results in relevant political environments by the Youth Harvest Foundation in association with the Swedish Association for Sexuality Education. The focus was on promoting CSE in schools in Ghana. In implementing the project, an approach focused on building trust and partnerships was used to broaden support for CSE’s content and also navigate political and cultural sensitivities regarding sexuality education (Entry Points Stage 1). This was done by shifting the focus from ‘human rights’ speeches to a strategic use of public health language to highlight its importance. Multi-stakeholder engagement was used to garner support and identify regional partners that would continue to support the programme (Entry Points Stages 4 and 5) [13, 14].

#### Case study 2: Establishing a Sexual Assault Care Centre (SACC) (Stages 0, 1, 4, 5)

The establishment of SACCs in Belgium involved a multi-stakeholder process that began with agenda-setting with policy-makers, as well as a systematic mapping of existing evidence and models (Entry Point Stage 0). This involved discussions in parliament, as well as engagement with policy-makers and experts, sustained over a long period of time. The results of a regional feasibility study served to convince policy-makers of the need to finance a national feasibility study. An output of the feasibility study, the development of a Belgian SACC model, was achieved due to the strong collaboration with different key stakeholders (politicians, service providers, police, justice, technical experts and survivors of violence). Trust building (Stage 1) among these stakeholders was key to the development of SACCs and to gain support for its pilot testing and implementation. Discussions and sharing of key findings occurred in round table discussions with different stakeholders. Media engagement was used as a way to disseminate findings and promote awareness and knowledge of these centres (Stages 4 and 5) [15, 16].

#### Case study 3: Providing comprehensive care to gender-based violence survivors (Stages 1, 4, 5)

This project involved the development and implementation of a framework for comprehensive care for sexual violence survivors in Kenya. This model involved the coordination of community-based responses, medical management of sexual violence and legal responses. The project focused on building trust by working with the Kenyan Ministry of Health, Health Institutions, non-governmental organisations (NGOs) in Kenya and the establishment of a steering committee for the project that involved representatives of all the relevant stakeholders (for example, the Kenyan police and women’s rights NGOs – Stage 1). Dissemination of key achievements was done using different media sources and multi-stakeholder meetings to promote support for the established centres in Kenya and encourage utilisation of the services by survivors (Stages 4 and 5) [17].

## Results: Findings and recommendations

The key themes that emerged from the facilitated group discussions are discussed herein. These recommendations were focused on two levels, namely researcher-stakeholder focused, where recommendations focused on ways to engage policy-makers and other stakeholders, and researcher-researcher focused, where recommendations focused on more efficient ways for collaborative research to promote the policy uptake of research.

These different types of recommendations were applied across the different stages identified that were relevant to the case studies. It is important to note that policy uptake of research findings is not a linear process and there are often time loops between stages. Nevertheless, to communicate the key findings of the workshop, we have described our findings using the stages described by Hanney et al. [1], specifically Stages 0, 1, 4 and 5, as these are opportunities within the policy formulation and implementation process for researchers to engage with stakeholders. An overview of the case studies and entry points are presented in Table 1.

**Table 1:** Summary of case studies

Focus of research	Region	Partners	Enabling factors	Disabling factors	Lessons learned	Opportunities
Instituting comprehensive sexuality education in schools	Ghana	International funders, academic institutions, local non-governmental organisations (NGOs)	Good multidisciplinary partnerships	Lack of political interest; backlash due to conservative views of programme	Understand the regional context and adapt key programmes and projects to acceptable language, that deliver the same quality but discourage backlash or conflicts with religious and traditional mores	Stages 1, 4 and 5
Establishing a sexual assault care centre	Belgium	Health ministries, teaching hospitals, academic institutions	Extensive background research on subject matter; multidisciplinary team, including service providers and politicians; extensive stakeholder engagement in all parts of the programme implementation process	Working with different political priorities and interests; this sometimes posed as a barrier for effective implementation	Sexual and reproductive health and rights researchers should create strong communication channels between themselves, policy-makers and other relevant actors to ensure that they are accessible and can be easily reached; this approach fosters dialogue and is strategic for promoting translation of research findings and outcomes into policies	Stages 0, 1, 4 and 5
Providing comprehensive care to gender-based violence survivors	Kenya	Ministry of Health, local NGOs, national hospitals and staff, international NGO and funders	Long-term partnerships with stakeholders; recognition as expert in the field; community engagement	Lack of resources and initial expertise or political interest	Sustained multi-stakeholder engagement was necessary over a long period for the development of trust, this enhanced the implementation of the project	Stages 1, 4 and 5

### Stage 0: Research needs assessment

#### Set the agenda (researcher-stakeholder focused)

Agenda-setting should be a role that researchers take on to promote policy uptake of their research findings. Researchers have the opportunity to steer policy in the direction of addressing key SRHR issues encountered in their work. Even though policy-makers might not view the issues identified as immediate priorities, engaging with policy-makers strategically provides an opportunity to contribute to setting the agenda and making it a priority issue. Opportunities should be identified for joint agenda-setting with policy-makers and other stakeholders. It is critical to engage and include the duty bearer of SRHR in the target country, as this institution or person will be the target for the policy action. An example of this was given by the case study on the establishment of the SACC in Belgium (Case study 2). At the beginning of the project, agenda-setting meetings with policy-makers and other stakeholders were held prior to the development of the model, providing an opportunity for researchers to engage policy-makers in dialogue and garner support among different stakeholders for the project implementation at the national level.

#### Align research to political priorities (researcher-stakeholder focused)

Researchers should make efforts to understand the SRHR political climate and frame research questions to strategically address these issues. For example, framing research within global priorities like the Sustainable Development Goals is strategic, as this is a priority for most policy-makers. They should also focus on planning research and advocacy activities and developing materials that are aligned with the political agenda and meet the information and evidence needs of stakeholders.

### Stage 1: Providing input to research development

#### Build trust and partnerships (researcher-stakeholder focused)

It is important to build trust with different stakeholders over time and not only at the point when there is a need to translate research findings into policy. The building of trust takes effort, multiple engagements and time investment. Developing ways for researchers to gain acceptance as experts that can provide technical input to policy formulation in policy dialogue is important. Similarly, developing respectful and equal partnerships between policy-makers and researchers is key. In Case study 1, while trying to improve comprehensive sexuality education in Ghana, an approach focused on building trust and partnerships through informal meetings and discussions with key policy-makers was used to broaden support for CSE’s content and also navigate political and cultural sensitivities regarding sexuality education.

#### Develop strong communication channels and pathways (researcher-stakeholder focused)

Researchers and policy-makers frequently speak different ‘languages’. It is therefore vital that researchers know the terms used by policy-makers and take advantage of informal meetings that provide opportunities for dialogue as well as for SRHR agenda- and priority-setting. Researchers should familiarise themselves with language and technical terms that policy-makers use to engage with them effectively. In cases where research findings may be unpopular, they should be conveyed in a manner that avoids conflicting interactions, while ensuring that research methods remain rigorous and research reports, devoid of bias. Where practically possible, the target institution or office for policy action should be involved in the generation of research findings. Engagements with stakeholders through informal meetings are opportunities to address misinformation and misconceptions about SRHR, whereas during formal meetings, policy-makers may be defensive, hindering the opportunity to discuss misinformation and misconceptions. Researchers should promote sustained communication channels with policy-makers. In Case study 1 (focused on comprehensive sexuality education in Ghana), this was achieved by avoiding ‘human rights’ speeches, as this was not effective for garnering support on comprehensive sexuality education in Ghana and instead the project team made use of public health discourses that focused on the health benefits of CSE. This strategy aligned to the popular political discourse and priorities in the context and encouraged support and uptake of the programme’s findings by policy-makers.

#### Multidisciplinary/interdisciplinary teams (researcher-researcher focused)

It is important for researchers to identify opportunities for developing multidisciplinary and interdisciplinary teams. Identifying ways of working together with different SRHR experts adds value to research and policy recommendations. For example, work on sexual violence should involve lawyers or social justice practitioners, human rights activists, the criminal justice system, health service providers and policy-makers. This is important during research development (Stages 0 and 1), as well as in the dissemination phase. In Case study 3, for example, in implementing a framework for comprehensive care for sexual gender-based violence survivors in Kenya, the project focused on building trust by working with the Kenyan Ministry of Health, Health Institutions, NGOs in Kenya and the establishment of a steering committee for the project that involved representatives of all the relevant stakeholders (for example, the Kenyan police and women’s rights NGOs).

#### Synthesise existing evidence (researcher-researcher focused)

SRHR researchers can optimise collaborative synthesis of existing evidence on a subject. By drawing on evidence across different contexts, countries and disciplines, they can develop stronger arguments for policy change based on best practices and implications for policy formulations. This type of evidence-based synthesis of research findings is more likely to be credible to policy-makers, than evidence from specific trials or cohorts, which do not make the linkage with policy development.

### Stage 4 and Stage 5: Dissemination of research findings and application of research findings

#### Multi-stakeholder engagement (researcher-stakeholder focused)

Multi-stakeholder engagement is important, allowing for a more holistic approach to translating research findings into policy and practice. This will provide opportunities for broader dissemination of research findings and uptake by practitioners. It is once again noteworthy that multi-stakeholder engagement should also occur at Stage 0. In all the case studies described above, engagement with different relevant stakeholders was crucial to the successful implementation of the projects, it provided opportunities to improve the project implementation process by getting the different stakeholders involved in proposing solutions, developing relevant action plans and assisting with the implementation process. A particular example of this is from Case study 2 (focused on the development and implementation of a Belgian model for SACC centres), wherein, during the agenda-setting stage, policy-makers, police officers, healthcare practitioners and other stakeholders were involved in consultation meetings to gain insights into how a comprehensive model could be developed to address challenges of sexual violence survivors accessing healthcare services.

#### Media engagement (researcher-stakeholder focused)

Media engagement is critical for disseminating research findings to the public. Strategic engagement with the media, policy-makers and advocates provides an opportunity to lobby for accessible translation of key SRHR research findings into policy and practice. Social media dissemination of research findings includes Twitter, Facebook, newspapers and radio stations; these also include agenda-setting opportunities. However, the use of these different types of media should be time and place relevant. In Case study 3 (focused on providing comprehensive response to sexual and gender-based violence survivors), dissemination of key achievements was done using different media sources and multi-stakeholder meetings to promote support for the established centres in Kenya and encourage utilisation of the services by survivors.

Researchers should be encouraged to evaluate and document good practices of translating research into policy. Activities like the ANSER workshop described in this article should be encouraged among different stakeholders, as this provides an opportunity to share and document good practices and lessons learned.

## Discussion

The findings from the participatory workshop and the literature review elucidate the importance of co-production and collaboration between researchers, policy-makers and other stakeholders to improve research utilisation in policy-making [18, 19]. This echoes similar findings in other research focused on uptake of SRHR research in policy-making [11]. The importance of identifying opportunities and strategic phases in the research and policy cycle where uptake of evidence could be maximised through dialogue with stakeholders is supported by evidence from the case studies discussed in our research in Ghana, Belgium and Kenya [14, 15, 17, 16], as well as by other research done in the Netherlands among stakeholders working in international development and SRHR [20]. Joint agenda-setting has been mentioned as an important approach for advocating the prioritisation of specific SRHR issues and an opportunity for policy framing, especially in low- and middle-income contexts [21]. Policy-makers often interpret their priorities through a context and value-laden lens. Understanding their decision-making process and the information sources that are valued the most by them, would strengthen researchers’ efforts to engage and advocate for specific SRHR priorities [10]. Developing strong communication pathways, skills and practices with policy-makers sustained over time, was found to be essential for the effective translation and dissemination of SRH research evidence by programme partners involved in developing a comprehensive care model for responding to sexual and gender-based violence in Kenya, as discussed in one of the case studies [17] and also among a health policy group in Nigeria [22]. An active engagement process, which involves sustained stakeholder engagement, dialogue with policy-makers, media engagement and pre-emptively synthesising relevant SRHR evidence, has been proposed by different research papers on SRHR policy [11, 23–26]. These papers echo our findings that the policy cycle is not linear, and sustained engagement is the best way to identify strategic entry points for policy uptake.

## Conclusions

Translating SRH research findings into feasible policy and practice is possible but needs to occur in conjunction with effective stakeholder engagement at different stages of the research cycle. This can only occur taking into account existing and changing political contexts and priorities. The ANSER is an opportunity to help close the gap between SRH research and policy.

### Key lessons

- Trust building is critical for translating research into policy or practice. However, trust building takes substantial but worthwhile time and resource investment. Building trust and fostering partnerships with policy-makers, service providers and other stakeholders should be a continuous process and not only at the point of research dissemination.
- Informal meetings provide an opportunity for researchers to network with stakeholders like policy-makers. There are many advantages gained from building trust and fostering partnerships between researchers and other stakeholders. These include, but are not limited to, increased uptake of research findings by different stakeholders.
- Researchers should engage with the media to ensure public dissemination of key research findings and emphasise key SRHR issues.
- Researchers should identify ‘knowledge gaps’ for policy-making and target their research to address these. Opportunities exist to develop an accountability framework between researchers and policy-makers. This can help in ensuring that health policies developed are evidence based and effective in addressing the most relevant problems and the most vulnerable populations.

## Appendix

### Guide used to facilitate the workshop sessions

Kolb et al.’s [12] experiential learning cycle has been used successfully in a myriad of adult learning processes and gives the base for bringing together the three dimensions of social learning and change (individual, organisational and societal/institutional) in a full spiral of action and reflection. Learning according to this theory involves a four-stage cyclical process. These four stages involve:

- Discussion of concrete experiences
- Opportunities for reflexive observations
- Abstract conceptualisation
- Concrete application

Key questions used to guide the discussions are outlined below:

1. What happened? What succeeded or failed? What significant things happened? Describe the events. Who was involved, what did they do?

How did stakeholders help/hinder this? What stakeholders? In what way?

2. Why did it happen? Why was it successful or not?

Why did it happen, what caused it? What helped, what hindered? What was expected? What assumptions were made? Are there other experiences or thinking that could help to view these experiences differently?

3. ‘So what’? What are the implications for the process?

What could have been done differently? What was learnt (new insights)? What new questions have emerged?

4. Now what? What action will we now take to make improvements?

What does this mean for practice? What is the goal, how should things change? What can be done differently? What is important to do in order not to repeat the same mistakes? What steps can be used to build these new insights into practice?

1. What happened? What succeeded or failed?

What significant things happened? Describe the events. Who was involved, what did they do? How did stakeholders help/hinder this? What stakeholders? In what way?

2. Why did it happen? Why was it successful or not?

Why did it happen, what caused it? What helped, what hindered? What was expected? What assumptions were made? Are there other experiences or thinking that could help to view these experiences differently?

3. ‘So what’? What are the implications for the process?

What could have been done differently? What was learnt (new insights)? What new questions have emerged?

4. Now what? What action will we now take to make improvements?

What does this mean for practice? What is the goal, how should things change? What is important to do in order not to repeat the same mistakes? What steps can be used to build these new insights into practice?

## Abbreviations

ANSER: Academic Network for Sexual and Reproductive Health and Rights Policy
CSE: comprehensive sexuality education
NGOs: non-governmental organisations
SACC: Sexual Assault Care Centres
SRH: sexual and reproductive health
SRHR: sexual and reproductive health and rights
